# Improving prevention and early detection of sepsis among patient groups at risk: Introducing a model for a multimodal information campaign—The SepWiss study protocol

**DOI:** 10.1371/journal.pone.0305107

**Published:** 2024-07-17

**Authors:** Wiltrud Abels, Konrad Reinhart, Edmund Neugebauer, Elisa Wulkotte, Evjenia Toubekis, Silke Piedmont, Sebastian Born, Thorsten Rieck, Odette Wegwarth, Claudia Spies, Peter Schlattmann, Daniel Schwarzkopf, Carolin Fleischmann-Struzek

**Affiliations:** 1 Department of Anesthesiology and Intensive Care, Charité – Universitätsmedizin Berlin, Berlin, Germany; 2 Sepsis Foundation, Berlin, Germany; 3 Brandenburg Medical School Theodor Fontane, Neuruppin, Germany; 4 Department for Infectious Disease Epidemiology, Immunization Unit, Robert Koch – Institute, Berlin, Germany; 5 Institute of Infectious Diseases and Infection Control, Jena University Hospital, Jena, Germany; 6 Max Planck Institute for Human Development, Berlin, Germany; 7 Institute for Medical Statistics, Computer and Data Sciences, Jena University Hospital, Jena, Germany; 8 Department for Anesthesiology and Intensive Care Medicine, Jena University Hospital, Jena, Germany; University of Zagreb School of Medicine: Sveuciliste u Zagrebu Medicinski fakultet, CROATIA

## Abstract

**Background:**

Sepsis is a life-threatening organ dysfunction due to a dysregulated host response to infection. Annually, sepsis leads to approx. 90.000 deaths in Germany. Risk factors include amongst others older age (>60), innate or acquired dysfunction of the immune system, and underlying chronic diseases of the lung, heart, liver, or kidneys. The manifestation of sepsis is a medical emergency, and patient outcomes depend on timely diagnosis and immediate treatment. In addition, vaccinations e.g., against pneumococci or influenza virus, are a highly effective public health tool to prevent the most common underlying infections that may lead to sepsis. However, a lack of public awareness for the relevance of vaccination and detecting sepsis as an emergency underlines the need for public health interventions that address these issues. SepWiss aims to evaluate the effects of a multimodal information campaign designed to address this lack of awareness among the risk population in Germany.

**Methods:**

SepWiss is an intervention at state level, consisting of a multimodal information campaign targeting risk groups in the German federal states of Berlin and Brandenburg (intervention region). Based on available evidence, various information formats were developed and implemented by outdoor advertising, social media, educational formats and through stakeholders’ platforms, starting in August 2021. The control region comprises of the remaining 14 German federal states. We will analyze vaccination coverage (primary outcome), and sepsis knowledge, the ability to detect sepsis as an emergency, and attitude towards vaccination (secondary outcomes) amongst the risk population in a controlled before-after comparison. The implementation is accompanied by a mixed-method process evaluation.

**Discussion:**

SepWiss is the first project of its kind to evaluate a complex multi-faceted evidence-based information campaign with regards to the topics of vaccination coverage, and the importance of sepsis detection and prevention for the most vulnerable populations in Germany. Results will be valuable for informing further nationwide campaigns.

**Trial registration:**

German Registry for Clinical Trials: DRKS00024475. Registered February 24^th^, 2021.

## 1 Background

Sepsis is life-threatening organ dysfunction due to a dysregulated host response to infection and can be caused by bacterial and viral organisms such as pneumococci, the influenza virus, or SARS-CoV-2 virus, as well as fungi or protozoa. Globally, 48.9 million incident cases of sepsis occur annually, resulting in 11 million sepsis-related deaths, or 20% of all-cause global mortality [1]. According to the WHO, a majority of sepsis cases would be preventable by improved hygiene, vaccinations, early detection, and timely and appropriate emergency treatment. The 2017 World Health Assembly resolution on “Improving the prevention, diagnosis and clinical management of sepsis” [2] urges member states to implement measures to increase public awareness around the risks of sepsis, and promotes the implementation of educational activities on recognizing sepsis as a preventable condition with a time-critical clinical course. In Germany, every year an estimated 240.000 patients are hospitalized with sepsis. Of these, 90.000 patients die [3].

Risk factors for developing sepsis include young (< 1) or older (> 60) age, underlying chronic conditions of the lung, heart or liver, diabetes mellitus, alcohol abuse, treatment with immunosuppressive medication, and asplenia. Patients with HIV or malignancies also have an increased risk of developing sepsis [4, 5]. Demographic change in Germany has led to an increasing number of patients at risk over the last decades. The Covid-19 pandemic impressively demonstrated how population groups at risk are vulnerable to a severe infection that can lead to sepsis. Risk factors for severe disease in Covid-19 are consistent with the risk factors for developing sepsis from other causes [6].

Vaccinations are highly effective public health tools to prevent infectious diseases and its complications including sepsis. Not only the COVID-19 vaccination but also others e.g., vaccination against pneumococci or influenza, have been recommended for patients with a high risk of infection or severe disease [7]. Nevertheless, recent studies amongst risk groups have shown that there is very little knowledge on how important vaccinations are to prevent sepsis [8]. In Germany, overall vaccination coverage of the adult population, particularly the elderly, is below other high-income countries, and thus worthy of improvement. In addition, individuals with vaccine-relevant pre-existing health conditions are often not fully vaccinated. E.g., in the 2021/22 Influenza season, influenza vaccination coverage was 43.3% among individuals aged 60 and older, and 35.4% among patients with pre-existing health conditions, and vaccination coverage for pneumococci was even lower with 23,3% and 25,6%, respectively [9]. It has been shown that increased sepsis knowledge might help to decrease vaccine hesitancy [10]. and that education of healthcare professionals might play an important in improving vaccine coverage [11]. The goal of increasing vaccine uptake is in line with the WHO sepsis resolution [2], which calls for prevention of the disease through the use of available vaccines.

In addition to vaccination, early detection of sepsis is critically important to reduce mortality and long-term sequelae. Reducing the time to start life-saving medications such as fluids and broad-spectrum antibiotics can reduce sepsis mortality [12, 13], and avoid deterioration of the patient and progression into septic shock [14]. Since a considerable percentage of sepsis cases are community-acquired [14], a better awareness for early symptoms and warning signs of sepsis is needed among the general population, similar to the common knowledge about the typical warning signs for acute myocardial infarction or stroke. As they are regarded as important sources of reliable health information [15], health professionals possibly act as multipliers of evidence-based information, leading to patients seeking more timely treatment.

The lack of awareness in the population at risk for the relevance of vaccination, as well as the lack of awareness for sepsis as an emergency underline the need for public health interventions that address these issues in an effective and sustainable manner. SepWiss therefore aims to design, implement, and evaluate a multi-faceted evidence-based information campaign with regards to the topic of vaccination coverage and the importance of sepsis prevention for the at-risk population in Germany.

### 1.1 Study objective

SepWiss is an intervention study on the design, implementation and evaluation of multimodal evidence-based sepsis awareness campaign targeting sepsis risk groups in Germany. We hypothesize that evidence-based information around the burden of sepsis, means to prevent it, and signs and symptoms for early detection might be able to foster informed choice, sepsis knowledge and ability to detect sepsis as an emergency in risk populations, and lead to improved vaccination coverage.

Thus, SepWiss aims to evaluate the effects of a multimodal evidence-based information campaign on

1. vaccination coverage amongst risk groups for pneumococci, haemophilus influenzae b, influenza virus and meningococcal infections (primary outcome), and
2. sepsis knowledge and ability to detect sepsis as an emergency, as well as attitude towards vaccination amongst the populations at risk (secondary outcomes).

In addition, SepWiss aims to identify facilitators and barriers to the implementation of a sepsis information campaign that may inform similar campaigns in the future.

## 2 Methods

### 2.1 Overview of the study design

SepWiss is an intervention study with a controlled before-after design. The intervention is a multimodal evidence-based information campaign about sepsis consisting of a) the development of evidence-based information formats made available at no costs, including information material and publications for existing platforms for health information in cooperation with multipliers, and a sepsis checklist; b) implementation of the campaign by large scale outdoor advertising, social media and (virtual) trainings for health care workers, family caretakers, and lay persons. The target population were risk groups for sepsis and sepsis-related mortality or morbidity. The intervention group were members of risk groups residing in the two German federal states of Berlin and Brandenburg, where the campaign was implemented from August 11, 2021. The control group was defined as members of risk groups residing in the remaining 14 federal states of Germany. The effects of the awareness campaign are assessed amongst risk groups through analysis of vaccination coverage (primary outcome), and assessment of sepsis knowledge, ability to detect sepsis as an emergency, as well as attitude towards vaccination (secondary outcomes) in a controlled before-after comparison with regards to the start of the information campaign. Vaccination coverage is assessed for the period from July 2016—June 2021 (pre intervention) and the period July 2021—December 2023 (post intervention start) based on administrative claims data of the associations of statutory health insurance physicians from outpatient care. Secondary outcomes are assessed via a structured survey among risk-groups in February 2021—July 2021 (pre intervention), February 2022—July 2022 (during intervention), March 2023—July 2023 (after intervention).

SepWiss is conducted by a consortium of researchers from the following institutions: Sepsis foundation, Max Planck Institute for Human Development Berlin (MPI), Charité–Universitätsmedizin Berlin, Brandenburg Medical School, Robert Koch–Institute (RKI), and Jena University Hospital. Trial sponsor is the Sepsis foundation. The authors conform that all ongoing and related trials have been registered with the study registry in the German register for clinical studies under the ID DRKS00024475. Data will be accessed by the respective partners conducting data assessment (for primary outcome: RKI; for secondary outcome: Jena University Hospital; for process evaluation: all members of the consortium).

**Fig 1:**
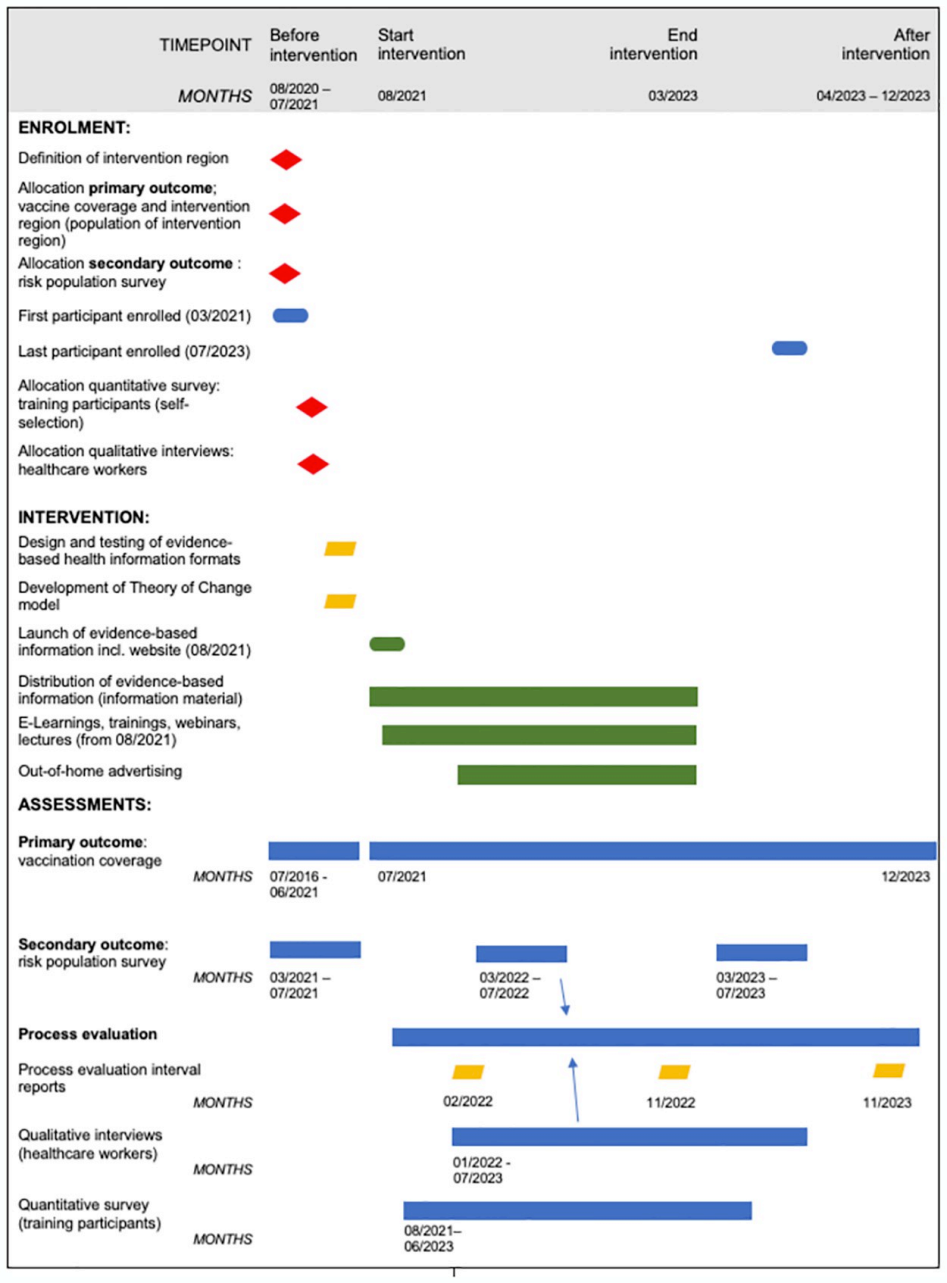
Overview flowchart modified from SPIRIT schedule. Overview flowchart of the SepWiss study, based on the SPIRIT guidelines [18] figure template; diamonds indicate decisions, ovals start or end points, parallelograms represent in-/outputs, rectangles present a process, arrows indicate an influence between respective shapes. Decisions on allocations and definitions are coded in red, outcome assessment and process evaluation elements are blue, outputs are yellow, and intervention elements green.

The description of the study follows the Transparent Reporting of Evaluations with Nonrandomized Designs (TREND) statement [16] (S1 File). An overview flowchart modified from the SPIRIT schedule template (Fig 1) and SPIRIT checklist (S3 File) are provided. Description of the intervention follows the Template for Intervention Description and Replication for population health and policy interventions (TIDieR-PHP) [17] (S1 File).

### 2.2 Ethics statement

Ethical approval for the SepWiss study was granted on December 16^th^, 2020, by the ethical committee of the University Hospital in Jena, Germany. In addition, ethical clearance for semi-structured interviews and a quantitative survey among healthcare workers was obtained from Charité (EA2/037/21; Berlin, March 30^th^, 2021) and Brandenburg Medical School (Z-01-20210723; Neuruppin, October 14^th^, 2021). Informed consent was collected from the study participants if participants were involved. This includes the risk group survey (secondary outcomes) and a quantitative and qualitative survey among healthcare workers. Informed consent was collected and saved at the respective centers, or at the market research institute IPSOS which conducted the risk group survey. Authors have no access to information that could identify individual participants during or after collection of quantitative data. Contrary to both surveys, the primary outcome is evaluated by the Robert Koch Institute (RKI) based on routinely collected pseudonymized claims data. The system is in regular use for analyzing vaccination coverage, based on the German Infection Protection Act as one of the core activities of the RKI. According to the ethical approval obtained for the study, individual informed consent was not necessary.

### 2.3 Target population

The campaign was targeted to reach the adult population with known risk factors for developing sepsis from an underlying infection. Amongst others, these include:

- older age (> 60 years)
- innate or acquired dysfunction of the immunosystem, such as immunodeficencies, immunopathies, HIV/AIDS
- long-term treatment with systemic immunosuppressive medication (e.g. for rheumatic disorders or autoimmune diseases)
- underlying chronic conditions of the lung, heart or liver
- asplenia
- diabetes mellitus

If one of these risk factors is present, vaccination against influenza and pneumococci is recommended according to the Standing Committee on Vaccination (STIKO); for asplenia, vaccination against haemophilus influenzae and meningococci is recommended [7].

### 2.4 Overall campaign strategy

In preparation of the design and implementation of the information campaign, qualitative piloting interviews with regional health professionals (general practitioners (GPs), emergency physicians, nursing director of a regional nursing home) were conducted between January and June 2021. These informed the development of a Theory of Change (ToC) model [19]. The ToC model served to refine the overall campaign strategy and to lay out assumed mechanisms in the sense of a backward mapping, starting from the desired long-term outcomes, i.e. improved vaccination coverage, improved sepsis knowledge amongst the risk population, and improved early detection and timely clinical management of sepsis. Based on this analysis, the following major strategies of the campaign were defined:

- Reaching risk-groups by addressing the general public: This approach was supported by the first survey amongst the risk population and previous research [15], in which family, friends and colleagues were identified as important sources of trustworthy health information. We furthermore identified other direct ways to address the population including e.g. the establishment of a sepsis information center on the campaign website, the provision of free information material, the use of social media, and partnering with advertising companies to realize outdoor advertising. In qualitative interviews, health professionals mentioned workload as a barrier to becoming the only active source of information around sepsis and expressed the perceived need for larger-scale public information activities.
- Reaching risk-groups by addressing health care professionals: As an indirect way of conveying information to the risk population, we identified the training of regional health professionals as an important intermediate outcome. This decision is backed up by a representative study that identified doctors as the most trusted source of health information for adult patients in Germany [15], and by findings that the continued education of healthcare workers can improve influenza vaccine uptake [11, 20]. Trained health professionals like GPs, pharmacists or nurses were assumed to be able to convey evidence-based information on vaccinations and sepsis to patients during their regular contacts with the risk population or their family members. To this end, different educational formats based on available evidence were planned and developed.
- Reaching risk-groups by addressing them or their relatives directly: Previous findings demonstrate that the most likely person to call an ambulance for someone else in case of a perceived emergency is a family member [21]. Additionally, a high proportion of patients receiving long-term care have an increased sepsis risk because of older age or underlying diseases, and the majority of long-term care is provided at home by relatives, not in nursing homes [22]. Based on this, we developed a targeted educational format for family members involved in caregiving for a relative and partnered with a large German health insurer to distribute information on sepsis for family caregivers in print and through an online platform.

Based on results of the accompanying process evaluation, the focus on the respective major elements of the campaign strategy was shifted over the course of the intervention. While in the first months of the campaign, involving and training health care providers, and distributing evidence-based information material were the major campaign strategies, initiating out-of-home advertising and addressing the risk population and their family members/caregivers directly became a stronger focus in the second half of the campaign.

### 2.5 Campaign organization

Implementing partners in the SepWiss consortium were the Sepsis Foundation, Charité and MHB. The team consisted of experienced senior researchers in the field of sepsis research, trained intensivists and physician consultants, social scientists and public health and health services research experts.

**Fig 2:**
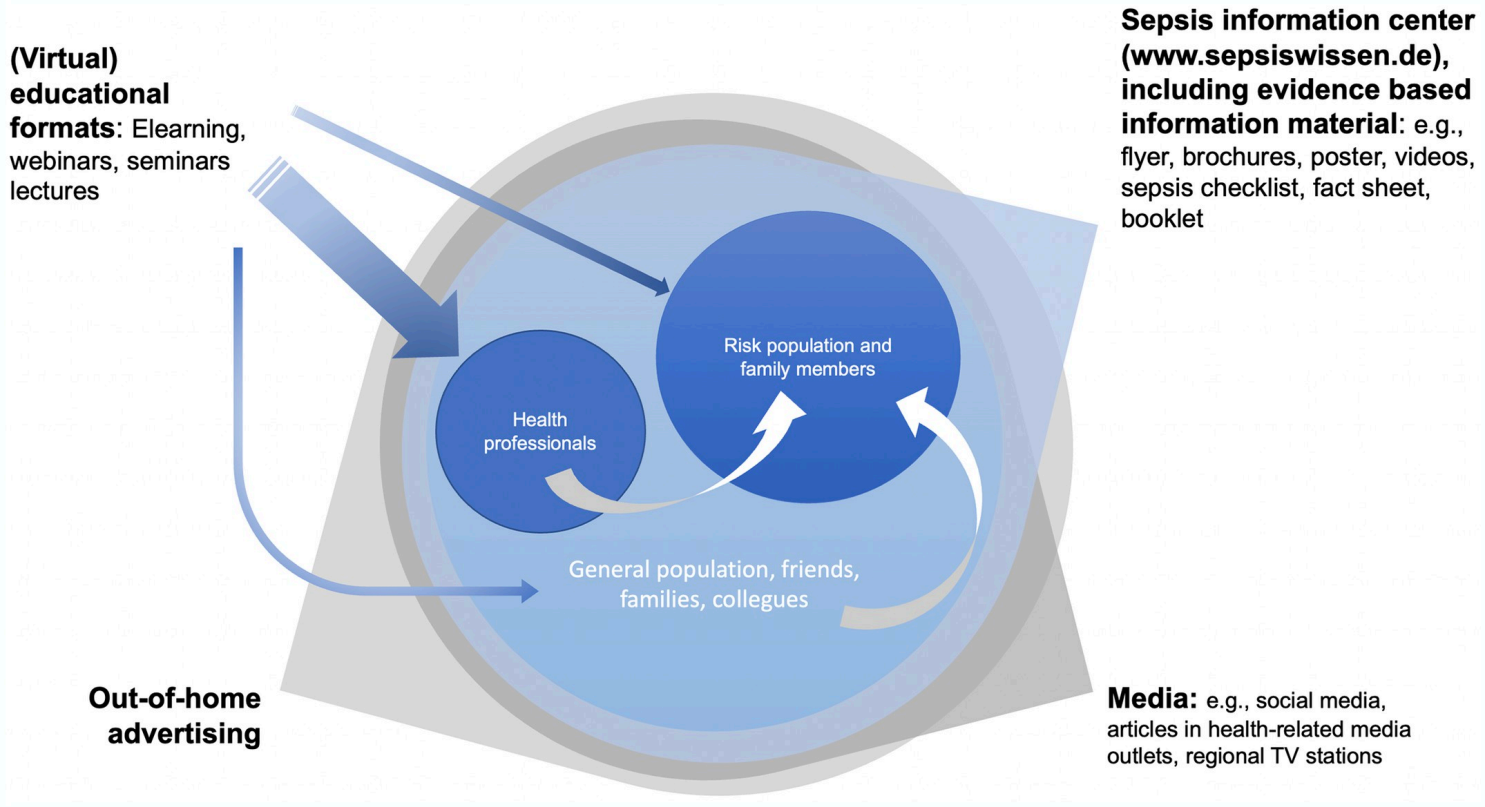
Elements of the multimodal information campaign. White arrows: assumed influence as preferred sources of health information.

Due to limited duration and funding of the project, involving regional stakeholders in distributing and multiplying SepWiss information was an essential element of the implementation from the planning phase onwards. Several stakeholders and organizations declared their willingness to support the SepWiss project through "letters of intent" (LOI) before the start of the campaign, including hospital operators, GPs and specialists in the primary healthcare sector, nursing facilities, health insurance companies, the Association of Statutory Health Insurance Physicians (German: Kassenärztliche Vereinigung), pharmacies, patient groups, and self-help organizations. Many of these were assumed to have good access to the risk population through established platforms and communication channels. Further LOIs were obtained from partners in the media and outdoor advertising sectors. To maximize outreach during the campaign, we contacted e.g., regional nursing homes, further regional hospitals, and regional professional associations of pharmacists, physicians, and nurses. The respective professional chambers of medical doctors and pharmacists in Berlin and Brandenburg became partners in hosting virtual trainings, providing their existing platforms for virtual as well as in-person lectures, talks and trainings. Nursing association, charities, and the state nursing council of Berlin and Brandenburg (German: “Landespflegerat Berlin-Brandenburg”) advertised the trainings and information material for nurses. Media outlets were contacted, and other interested parties, e.g., regional banks, became supporters. Many activities were realized with support from stakeholders and supporting organizations. From the start, establishing and continuously growing a network of regional supporters was thought to be a prerequisite to successful implementation. Intervention elements are depicted in Fig 2.

### 2.6 Intervention elements

#### 2.6.1 Development of evidence-based educational content

To inform all elements of the information campaign, available information on sepsis prevention and early detection was identified in a systematic rapid literature review and evidence synthesis in the first half of 2021 [23]. Based on this information, two different information formats (graphic vs. text-based) were developed and tested in a survey among risk groups for acceptability and impact on informed decision-making by the consortium partners at Max Planck Institute of Human Development. Details of the survey study have already been published elsewhere [24]. After this development phase, materials were continuously updated and developed further with regards to content and format by the implementing partners. Expert-informed printed and electronic versions of educational content like brochures, flyers and posters were developed and made available for free from the project website (see 2.6.2 Central resources).

As another educational tool, a sepsis checklist was developed by the implementing partners in order to support the decision to call for help in case of suspected sepsis. Content of the checklist was made available from the project website.

All educational materials are based on the available evidence identified by the partners at Max Planck Institute, as well as expertise of researchers and clinicians with long-standing experience, and international experts in the field of quality improvement of sepsis care. Formats were also developed further in comparison to materials used by awareness campaigns in the US, UK and Australia.

#### 2.6.2 Central resources

In the preparatory phase of the information campaign’s implementation, the website www.sepsiswissen.de was established as an information center on sepsis and vaccine-preventable infections, offering information materials, frequently asked questions, news, scientific and other publications for the general public including the risk population, health-care workers, media and other stakeholders. A separate section “COVID-19 and Sepsis” was introduced in the light of the ongoing pandemic at the time. The website went online in July 2021, just before the start of the implementation. Information on available training formats and dates, as well as an option to download and/or order free evidence-based information material was included and updated in regular intervals. Page visits and usage of the site are quantified with the Google Analytics^®^ tool throughout the project.

#### 2.6.3 Elements to address the general public

In cooperation with two regional companies for large scale out-of-home advertising, we developed information formats that were displayed on outdoor poster formats in Berlin and Brandenburg over the course of different weeks in 2022. Further cooperation was established with platforms for advertising in public transport in Berlin. Information material was distributed to all pharmacies in Berlin and Brandenburg (> 700 in Berlin and > 2000 in Brandenburg) in cooperation with the pharmacist associations of both federal states. SepWiss was additionally promoted in cost-free pharmacy magazines widely read by laypeople. Implementing partners established cooperation with regional banks in Brandenburg that displayed information about SepWiss on the screens of ATMs as well as during online banking transactions. Information material was available from several branches of in Brandenburg, and employees were trained to promote the information material. Information was also spread by social media via the channels of the consortium on Facebook, Twitter, LinkedIn and Instagram. Several articles were published in health-related online media outlets as well as regional print media, and the project was reported about by a regional TV station.

#### 2.6.4 Elements to reach health care professionals

*2*.*6*.*4*.*1 Journal articles*. More than ten non-peer reviewed articles authored by the implementing partners from Sepsis Foundation, Charité and MHB were published in health professional journals and on websites of regional professional associations of pharmacists, physicians, and nurses. These publications served to promote the SepWiss training formats, as well as raise sepsis awareness amongst health professionals.

*2*.*6*.*4*.*2 (Virtual) educational formats*. SepWiss provided several different educational formats to achieve the intermediate outcome of training health professionals. These formats were offered over the whole course of the intervention. Various formats of similar content were planned for different professional groups. The basic topics remained unchanged, including the primary prevention by recommended vaccines and early detection of sepsis. At the same time, formats were adjusted to the targeted group, and enriched by specified themes, e.g., on prehospital sepsis recognition and on the connection of sepsis and COVID-19 in the light of the ongoing pandemic. Formats included virtual learning in e-learning modules (online on-demand, 45 minutes per module), interactive webinars (online live, 30 minutes to 1.5 hours), live lectures, in-person seminars, and workshops (both live and in-person) for GPs, other physicians, pharmacists, and nursing staff. Training formats were delivered by experienced senior researchers, physician consultants and health scientists from the Sepsis foundation, Charité, and MHB. Participants self-registered online on the project website or on respective multipliers’ platforms for E-Learnings and webinars. As an incentive, participants were able to claim credits for continued medical education (CME) for taking part in webinars and E-Learnings. As a planned variation, formats were continuously developed further in a targeted way e.g., to be used on pre-existing platforms of partnering stakeholders.

With the limitations of the COVID-19 pandemic in place as an important contextual factor, evidence-based information was initially translated into largely virtual training formats for healthcare professionals. When the limitations of the ongoing COVID-19 pandemic subsided, in-person lectures and presentation formats became more frequent.

#### 2.6.5 Elements to directly reach members of the target populations or their relatives

After the first round of process evaluation, virtual and non-virtual learning formats for risk groups and family members were added to address the target population more directly. Short video formats were developed for the purpose of being displayed in the waiting areas of hospitals or doctor’s offices and implemented in several waiting areas at Charité University Hospital. In cooperation with a large health insurer, we targeted family caregivers with educational material for family caregivers and short information formats for publication on a dedicated online platform and to be handed out to laypeople in seminars (open to people with any health insurance).

None of the mentioned intervention elements were expected to result in any adverse events or harm for recipients or participants. All members of the consortium participated in a planned process evaluation (see 2.9 Process evaluation–Evaluation of the implementation), including interim analyses of available data and the option to adapt the implementation strategy.

### 2.7 Implementation of the campaign

Start date for the implementation of the SepWiss campaign was August 11^th^, 2021. Not all elements of the campaign could be finalized and implemented with the launch of the campaign. The ongoing COVID-19 pandemic necessitated an initial focus on developing virtual educational formats in the first half of the campaign, and in-person formats only became possible when contact restrictions due to the pandemic subsided. In addition, some elements of the campaign could not be implemented before additional stakeholders like regional media outlets and professional associations had been contacted and involved. Thus, the different elements of the campaign were implemented consecutively, as depicted in Fig 1.

### 2.8 Evaluation of the intervention

#### 2.8.1 Assessment of the primary outcome

Primary outcome will be assessed by the partners at German public health institute Robert Koch–Institute (RKI) in Berlin.

We hypothesize that the intervention will have a positive impact on vaccination coverage of influenza, pneumococci, meningococci and haemophilus influenzae type b vaccination in the target population, and thus vaccination coverage will increase during the intervention period and will be higher in Berlin and Brandenburg (intervention region) compared to other federal states in Germany (control region). To test this hypothesis, vaccination coverages for aforementioned vaccinations are analyzed.

*2*.*8*.*1*.*1 Data source and population*. Data base are all outpatient claims data of all statutory health insured (SHI) individuals in Germany, covering approximately 87% of the general population. These data are routinely reported to RKI, and no sample size calculation was needed. The data set includes information about month and year of birth, sex, federal state of residence, type and date of vaccination and International Statistical Classification of Diseases (ICD-10) coded diagnoses. Detailed information on the data set is provided elsewhere [25].

Vaccination coverage can be calculated for influenza, pneumococci, meningococci and haemophilus influenzae type b vaccination in three different target populations based on the vaccination recommendations by the National Immunization Technical Advisory Group in Germany: (a) the elderly population aged 60 and above, (b) pediatric population aged three years and younger, and (c) population with vaccine-relevant pre-existing health conditions.

*2*.*8*.*1*.*2 Outcomes*. Outcome measures include vaccine coverage among the three target groups before / early in the intervention versus during its full implementation. Data from Berlin and Brandenburg will be analyzed versus the other federal states of Germany (control group). Data from the pre-intervention period (July 2016 until June 2021) will be compared to data from the post-intervention start period (July 2021 until December 2023).

*2*.*8*.*1*.*3 Statistical analyses*. We use a cohort approach to calculate vaccination coverage. This enables age-, sex- and region-specific analyses as well as an analysis of vaccine coverage in consideration of a health indication to vaccinate [25]. Vaccination coverage is calculated as the proportion of individuals among a cohort who received the vaccine within the influenza season (influenza or pneumococci) or as a single dose as an adult (meningococci and haemophilus influenzae type b). The results are reported as vaccination coverage per season and calendar year, respectively.

#### 2.8.2 Assessment of the secondary outcome

Secondary outcome is assessed by the partners at the Jena University Hospital in a longitudinal survey of risk groups.

*2*.*8*.*2*.*1 Data source and sample*. The survey was conducted at three measurement points before, during, and after the implementation of the information campaign, in March—July 2021 (before), March—July 2022 (during) and March—July 2023 (after). All parts of the survey were conducted both in the intervention regions and the remaining German states as the control condition. The evaluation of secondary outcomes is based on the first and third time point, while the second time point served to inform the process evaluation of the campaign (see 2.9.).

A convenience sample of *n* = 740 persons with a minimum age of 18 years was recruited by the market research institute IPSOS. The recruitment was designed to achieve predefined numbers of cases within strata defined by age (<60 years, ≥60 years), education (low, middle, high), specific chronic diseases (e.g., diabetes, chronic diseases, cancer), and regions (intervention vs. control states). Sample recruitment for persons without chronic diseases was realized via an existing consumer survey panel. Sample recruitment for the group of persons with specific diseases, sample recruitment was carried out via self-help groups, general practitioners, social media and by contacting participants of previous studies by IPSOS. After they gave informed consent to participate in the survey study, participants were interviewed online, via telephone or face-to-face, using a standardized questionnaire in German language.

*2*.*8*.*2*.*2 Sample size considerations*. As the sample size required for the analysis depends on a number of factors (e.g. stability of the outcomes between the survey time points, variance of the outcomes at the survey dates, the intervention effect, which cannot be specified precisely in advance), several scenarios were considered as part of a simulation study. The power simulation was conducted in the statistical software R [26] version 4.0.2) with the R package lme4 [27]. As result a sample size of *N* = 244 for the third survey time point seems to be sufficient for detecting a moderate effect (*d* = 0.5) at a significance level of α = 0.05 with a power of 80%, even with low stability of the outcome variables (correlation of *r* = 0.3 between survey time points). However, the resulting sample size is only sufficient under the assumption that there are no missing values on relevant variables. To allow for a reduction in the effective sample size due to missing values, the sample size estimated from the simulation study was increased by 15%, resulting in a sample size of N = 280.

A necessary condition for the planned analyses and the power simulation carried out is the participation at both, first and third, survey time points. For this reason, the dropout rate must be taken into account for the sample size consideration. Based on the experience from the market research institute a drop-out of about 60% from the first to the third survey time point was used, resulting in a total sample size of *N* = 740 for the first time point.

*2*.*8*.*2*.*3 Outcomes*. We developed a questionnaire to assess sepsis knowledge, ability to detect sepsis as an emergency, and attitude towards vaccination (secondary outcomes). Sepsis knowledge was assessed in the following five domains using an adaption and extension of an existing instrument (8): definition and epidemiology (12 items), general prevention (3 items), symptoms (7 items), risk factors (7 items), and sepsis-specific vaccination knowledge (7 items). To measure the ability to detect sepsis as an emergency, five case vignettes were developed including typical sepsis-related medical emergencies (e.g., sepsis due to urinary tract infection or sepsis due to respiratory infection), for which participants had to rate how they would react in the described situation using an ordinal three-step-scale (0—“Wait one day and decide then”, 1—“Visit the general practitioner on the same day”, 2—“Call the emergency service or go to the emergency department”). Vaccination behavior and attitude towards vaccination was assessed based on the Theory of planned behavior [28] and the 5C-model [29]. For the above-mentioned outcomes, an item pool and first draft of the questionnaire was created. Content validity, relevance and comprehensibility of the questionnaire were evaluated by cognitive interviews with experts in the field of emergency medicine, infectious diseases, intensive care medicine and psychiatrics (n = 5), and one patient, and the questionnaire was revised based on the results. A pretest of the revised questionnaire was conducted in a pilot survey among members of patient support groups for chronic diseases (n = 71) and item characteristics as well as number of missing responses were investigated. Based on the results of the pre-test, we modified minor details of the questionnaire to receive the final draft. The questionnaire was administered in German language.

*2*.*8*.*2*.*4 Statistical analysis*. To assess the effect of the intervention in the controlled before-after design, a linear mixed model will be calculated with the variables survey time point (first and third time point) and region (intervention and control region). The hypothesis of campaign effectiveness is examined by testing the interaction effect between survey time and region against zero. Survey time point, region and the interaction effect are estimated as fixed effects. Systematic differences between persons are taken into account as random effect. Differences in confounders between the control and intervention regions are adjusted by including the following confounders as covariates in the model: presence of chronic disease, age, gender, education level, health information seeking behavior (frequency of health information seeking and variety of information sources). Since 15% of missing values were already taken into account in the sample size consideration, list wise deletion is used in the analysis to deal with missing values. The analyses will be conducted with statistic software R and the R package “lme4” with a significance level of α = 0.05.

### 2.9 Process evaluation—Evaluation of campaign implementation

**Fig 3:**
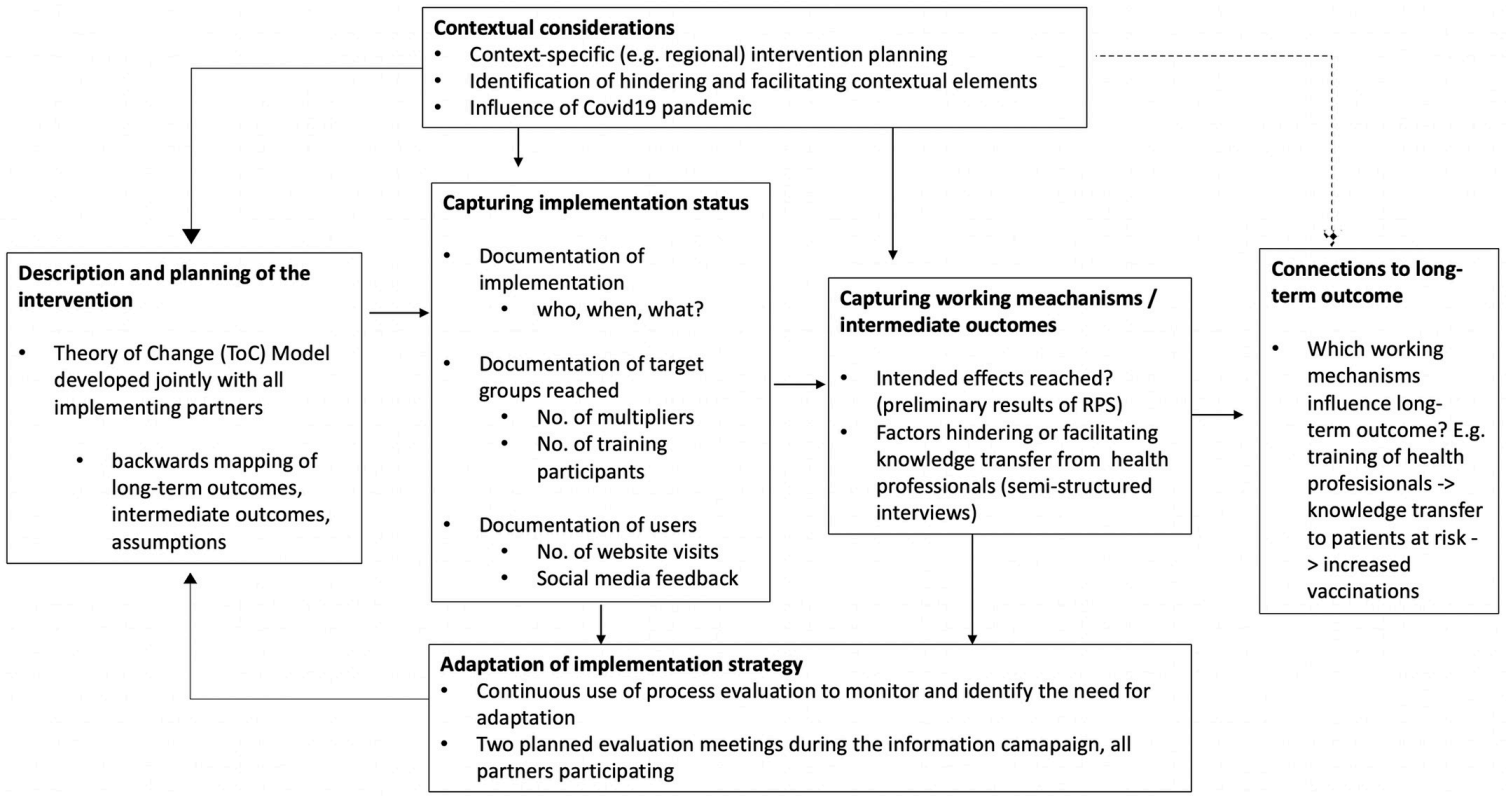
SepWiss process evaluation. RPS = risk population survey.

To ensure replicability and transferability of complex interventions to other contexts, an accompanying process evaluation that assesses feasibility and success of implementation, mechanisms of action, and influencing contextual factors has been recommended [30, 31]. In accordance with this guidance, a prospective process evaluation for the implementation of the SepWiss information campaign was planned, including the elements shown in Fig 3.

The process evaluation involves all partners of the consortium and serves to 1) support the implementation planning, 2) monitor implementation status and, 3) guide adjustment of implementation strategy, if necessary.

Planned indicators used to inform the process evaluation were e.g., the number of page views on the project website in connection to campaign activities, intermittent analyses of vaccination coverage, results from the three risk group surveys, and results of semi-structured qualitative interviews and quantitative surveys with health professionals actively involved in patient care in Berlin or Brandenburg. These interviews served to identify factors that hinder or facilitate health care professionals’ willingness and ability to increase risk groups’ sepsis knowledge, and to analyze which component of the campaign are used by health care professionals to support their patients’ knowledge.

As an unpredictable force majeure, the at the time ongoing Covid-19 pandemic was taken into account at every step of the process evaluation. Analyses of vaccination coverage from 2021/2022 did not show a negative impact on the uptake of standard vaccinations, but were able to prove positive trends e.g., higher vaccination coverage for pneumococci vaccination in the beginning of 2022 compared to 2019 [32]. Eventually, potential effects of the COVID-19 pandemic on the results of the SepWiss study such as vaccination fatigue post COVID-19 will be discussed in more detail upon publication of the results.

## 3 Results

### 3.1 Current state of the study

The SepWiss information campaign activities started in August 2021. After March 31, 2023, there are no further activities directed at the general public. Educational formats are not actively advertised anymore but are provided upon request. Information materials and recorded formats remain available from the information center. Data collection for assessment of vaccine coverage (primary outcome) and evaluation of the risk group survey (secondary outcomes) have not been finished yet. The evaluation of campaign effects will be ongoing until spring 2024.

## 4 Discussion

SepWiss evaluates the effects of a multi-faceted evidence-based information campaign on vaccination coverage of recommended vaccines as well as sepsis knowledge within the most vulnerable populations in the German federal states of Berlin and Brandenburg. The SepWiss campaign is unique in its scope and design. Previous regional awareness campaigns, like the project "MV vaccinates" (German: “MV impft”) in the German state of Mecklenburg-Vorpommern, did not emphasize the need for evaluating campaign success, and furthermore did not emphasize the element of potentially preventing sepsis by avoiding vaccine-preventable infections. Some of the partners from the SepWiss consortium were involved before in the BMBF-funded project "Vaccination 60+" (German: “Impfen 60+") in the German state of Thuringia, which included an evaluation of an intervention to increase vaccination coverage, but, as a notable difference to SepWiss, did not address the topic of early sepsis detection. Also in international comparison, SepWiss stands out by evaluating the impact of targeted health information on sepsis knowledge as well as vaccination coverage. High-profile nationwide sepsis awareness campaigns have been driven for a long time now by the CDC and the Rory Staunton Foundation in the US, or the UK Sepsis Trust and National Health Service in the UK, respectively. However, none of these are focused on specific risk groups, nor are they closely linked to the issue of vaccine prevention.

The study has important strengths, including its evidence-based design, the multimodal intervention and evaluation strategy, the involvement of public health stakeholders, and the controlled before-after design. However, the evaluation of outcomes, in particular vaccination coverage and attitudes to vaccination, might be influenced by well-known factors such as trust in vaccine efficacy, barriers in access, or risk perception [29], not all of which are specifically addressed and measured in the study. In addition, the effects of the COVID-19 pandemic as an unpredictable force majeure at the time of implementation could not have been addressed in advance by the design of the evaluation of the primary and secondary outcomes. However, the before-after design including control regions still allows to analyze effects in the intervention region. In addition, the planned process evaluation was adapted and carried out in more depth to identify the campaign’s impact mechanisms in the light of the ongoing pandemic. The potential effects of the COVID-19 pandemic at the time of campaign implementation will be discussed along with the reporting of study outcomes.

The SepWiss project helps to better understand which measures are suitable in the German healthcare system to increase vaccination coverage against sepsis-causing infections and to improve the early detection of sepsis. The findings of the project can make a significant contribution to implementing the requirements of the WHO resolution "to include prevention, diagnosis and treatment of sepsis in national health systems strengthening in the community and in health care settings" [2] at the federal level. Taking appropriate measures was also proposed in a unanimous resolution of the 2018 German Conference of Health Ministers [33]. The multimodal SepWiss campaign addresses the insufficient vaccine uptake among people with underlying diseases and among the elderly in Germany [25] by trying to increase sepsis knowledge among the risk population, which has been shown to positively influence vaccine hesitancy [10], as well as train health care workers, which potentially leads to improved vaccine uptake [11]. With this, the study contributes to protecting the population from vaccine-preventable diseases. It also supports the recommendations from the European Council to aim for an influenza vaccination coverage of 75% among people > 60 years of age [34], which is also part of the German national vaccination strategy [35]. Evaluating the campaigns effects in the metropolitan region of Berlin as well as in the more rural state of Brandenburg, results are expected to be particularly valuable in informing nationwide sepsis awareness activities in the future.

## Supporting information

S1 File TREND and TiDier checklists. (DOCX)

S2 File SPIRIT checklist. (DOC)

S3 File Original study protocol (German). (PDF)

## Abbreviations

GP: general practitioner
LOI: Letter of Intent
MHB: Brandenburg Medical School (German: Medizinische Hochschule Brandenburg)
RPS: risk population survey
STIKO: Standing Committee on Vaccination (German: Ständige Impfkommission)
ToC: Theory of Change
