# Supplementary material for: Improving prevention and early detection of sepsis among patient groups at risk: Introducing a model for a multimodal information campaign—The SepWiss study protocol
Source: PLoS One. 2024 Jul 17;19(7):e0305107. doi: 10.1371/journal.pone.0305107 (PMC11253930; doi:10.1371/journal.pone.0305107)
Supplement: S3 File — (PDF) [file pone.0305107.s003.pdf]

Studienprotokoll

Stärkung der Gesundheitskompetenz von Sepsis-Risikogruppen zur Verbesserung der Sepsisfrüherkennung und -prävention (SepWiss)

Protokollversion:	Entwurfsversion 17.07.2020
Vertraulichkeitshinweis:	Der Inhalt vom vorliegenden Studienprotokoll ist vertraulich zu behandeln und darf ohne Zustimmung vom Studienleiter weder mündlich noch schriftlich an Unbeteiligte weitergegeben werden.

Inhaltsverzeichnis

1. ALLGEMEINE INFORMATIONEN 4

2. HINTERGRUND 6

3. STUDIENZIELE 7

4. STUDIENDESIGN UND -BESCHREIBUNG 8

4.1. Art der Studie 8

4.2. Einschlusskriterien und Rekrutierung 8

4.3. Ablauf der Studie 10
4.3.1. Arbeitspaket 1: Entwicklung der Informationsformate 10
4.3.2. Arbeitspaket 2: Durchführung der Informationskampagne 10
4.3.2.1. Multimodale Implementierungsstrategie der Kampagne 10
4.3.2.2 Prozessevaluation 11
4.3.3. Arbeitspaket 3: Evaluierung der Informationskampagne durch Auswertung der Impfquoten in den Risikogruppen 13
4.3.4. Arbeitspaket 4: Evaluierung der Informationskampagne durch Befragung von Risikogruppen in der Modellregion 13

4.4. Anzahl der zu Befragenden und Biometrie 14
4.4.1. Arbeitspaket 1 14
4.4.1.1. Messungen 14
4.4.1.2. Fallzahlkalkulation 14
4.4.1.3. Statistische Analysen 15
4.4.1. Arbeitspaket 2 15
4.4.2. Arbeitspaket 3 15
4.4.2.1. Messungen 15
4.4.2.2. Statistische Analysen 16
4.4.3. Arbeitspaket 4: Befragung von Risikogruppen 16
4.4.3.1. Messungen 16
4.4.3.2. Fallzahlkalkulation 17
4.4.3.3. Statistische Analysen 17

4.5. Zeitplan 17

5. TEILNEHMENDE EINRICHTUNGEN 18

6. DATENMANAGEMENT 20

7. ETHISCHE BELANGE, GESETZLICHE UND ADMINISTRATIVE REGELUNGEN 21

7.1. Deklaration von Helsinki und Gute klinische Praxis 21

7.2. Ethik-Kommissionen 21

7.3. Nachträgliche Änderungen 21

8. FINANZIERUNG 22

9. ABSCHLUSSBERICHT UND PUBLIKATION 22

10. LITERATUR 23

1. Allgemeine Informationen

Name	Institution	Telefon, Fax, E-Mail	Verantwortlichkeit/Rolle
Prof. Dr. K. Reinhart	Sepsis-Stiftung	Tel. +49 30 450 551 415<br>E-Mail: konrad.reinhart @charite.de	Studienleitung/ Konsortialführung
PD Dr. O. Wegwarth	Max-Planck-Institut für Bildungsforschung, Berlin (MPIB)	Tel. +49 30 82 406 695<br>Fax +49 30 82 406 394<br>E-Mail: wegwarth@ mpib-berlin.mpg.de	Arbeitspaket 1: Entwicklung und Evaluation evidenzbasierter Informationsmaterialien für die Informationskampagne
PD Dr. C. Hartog,<br>Prof. Dr. Claudia Spies	Charité Berlin (CUB)	Tel. +49 30 450 531 089<br>Fax +49 30 450 531 911<br>E-Mail: Christiane.hartog @charite.de, Claudia.spies@charite.de	Arbeitspaket 2: Kampagnenimplementierung, Evaluierung der Interaktion mit Multiplikatoren
Prof. Dr. E. Neugebauer	Medizinische Hochschule Brandenburg (MHB)	Tel. +49 3391 39141 10<br>Fax +49 3391 39141 09<br>E-Mail: Edmund.neugebauer @mhb-fontane.de	Arbeitspaket 2: Kampagnenimplementierung, Evaluierung der Interaktion mit Multiplikatoren
PD Dr. O. Wichmann	Robert-Koch-Institut (RKI)	Tel. +49 30 18754 3468<br>Fax +49 30 18754 3572<br>E-Mail: WichmannO@rki.de	Arbeitspaket 3: Kampagnenevaluation: Impfquoten und Impfinzidenzen
Dr. C. Fleischmann-Struzek,<br>Dr. D. Schwarzkopf	Center for Sepsis Control & Care, Univ.-Klinik Jena (UKJ)	Tel. +49 3641 9323 146/-195<br>Fax +49 3641 9323 379<br>Email: Carolin.fleischmann @med.uni-jena.de, Daniel.schwarzkopf@ med.uni-jena.de	Arbeitspaket 4: Kampagnenevaluation: Befragung von Personen aus Risikogruppen
Prof. Dr. P. Schlattmann	Institut für Medizinische Statistik, Informatik und Dokumentation, Univ.-Klinik Jena (UKJ)	Tel. +49 3641 934 130<br>Fax +49 3641 933 200<br>E-Mail: peter.schlattmann@ med.uni-jena.de	Hauptverantwortlicher Biometrie

Unterschriften

[redacted]

11.08.2020

Prof. Dr. Konrad Reinhart, Konsortialführung — Datum

[redacted]

25.08.2020

PD Dr. Odette Wegwarth, MPI f. Bildungsforschung — Datum

[redacted]

tt.mm.2020

31.08.20

Prof. Dr. [redacted]arité Berlin — Datum

[redacted]

11.08.2020

Prof. Dr. Edmund Neugebauer, MHB — Datum

[redacted]

31.08.2020

PD Dr. Ole Wichmann, RKI — Datum

[redacted] 11.08.2020

Dr. Daniel Schwarzkopf, UKJ Datum

[redacted] 11.08.2020

Dr. Carolin Fleischmann-Struzek, UKJ Datum

[redacted] 25.08.2020

Prof. Dr. Peter Schlattmann, Biometriker, UKJ Datum

2. Hintergrund

Die WHO hat in der 2017 von der World Health Assembly verabschiedeten Resolution „Improving the prevention, diagnosis and clinical management of sepsis" festgehalten, dass die Mehrzahl der Sepsisfälle vermeidbar ist und es deshalb notwendig ist, die Awareness für Sepsis zu steigern [1]. In einem von zahlreichen Fachgesellschaften und Experten unterstützten Memorandum für einen Nationalen Sepsisplan wird geschlussfolgert, dass durch die Umsetzung einfacher, kosteneffektiver Maßnahmen allein in Deutschland jährlich 15.000-20.000 Todesfälle durch Sepsis vermeidbar sind (https://www.sepsis-stiftung.eu/wp-content/uploads/1/2018/11/2018_04_01_Memorandum_Sepsisplan_gesamt.pdf). Laut einer repräsentativen Umfrage wissen aber nur 17% der Deutschen über 60 Jahre, dass Sepsis häufig durch Infektionen ausgelöst wird, gegen die man sich durch Impfungen schützen kann [3]. Dabei ist die Evidenz für die Effektivität von Impfungen gegen Pneumokokken, Meningokokken, Influenzaviren und Haemophilus influenzae Typ b (Hib), welche die häufigsten Auslöser einer Sepsis sind, hoch [4, 5]. Pneumokokken sind die häufigsten Erreger der ambulant erworbenen Pneumonie. Pneumonie ist sowohl die häufigste Ursache für eine ambulant erworbene Sepsis als auch die häufigste Ursache für infektionsbedingte Hospitalisierungen in Deutschland [4]. Die Impfquote gegen Pneumokokken ist in Deutschland

mit 31,4% jedoch deutlich niedriger als in den USA (63,6%), England (69,8%) und Australien (56,0%) [2]. Auch die Impfquote gegen Influenza ist in Deutschland mit 31,4% niedriger als in den USA (69,1%), England (71,1%) und Australien (74,6%). Insbesondere für Sepsis-Risikogruppen, denen etwa 34,5 Mio. Bundesbürger zuzurechnen sind, gibt es spezifische Impfempfehlungen der Ständigen Impfkommission (STIKO) am RKI [6]. Als Risikogruppen gelten Menschen über 60 Jahre, Schwangere, Menschen mit angeborener oder erworbener Fehlfunktion des Immunsystems (u.a. bei HIV/AIDS), Menschen ohne Milz, Patienten unter Langzeittherapie mit immunsupprimierenden Medikamenten (z.B. wegen rheumatischer Erkrankungen, Autoimmunerkrankungen, schwerer Psoriasis), Menschen mit Krebs, Menschen mit chronischen Erkrankungen des Herzens, der Lunge, der Leber, der Niere oder Diabetes mellitus, Alkohol- bzw. Drogenabhängige.

Neben der Impfprävention kommt der Früherkennung der Sepsis eine entscheidende Rolle zu, da die Reduzierung der Zeit bis zur Einleitung evidenz-basierter Therapiemaßnahmen bei Sepsis die Sterblichkeit um 1-2% pro Stunde vermindert [9, 10].

Im Rahmen der vorliegenden Studie sollen evidenzbasierte Gesundheitsinformationen zur Früherkennung und Prävention von Sepsis für Risikogruppen entwickelt und mittels einer multimodalen Informationskampagne in den Interventionsregionen Berlin und Brandenburg unter Beteiligung wesentlicher Akteure im Gesundheitswesen, Interessen- und Selbsthilfegruppen sowie Medien verbreitet werden. Auf diese Weise soll die Gesundheitskompetenz von Risikogruppen für Sepsis verbessert und eine Erhöhung der Impfquote gegen eine Sepsis-auslösende Infektionskrankheit wie Grippe, Lungen- oder Hirnhautentzündung erreicht werden.

3. Studienziele

Primäres Ziel der Interventionsstudie ist die Untersuchung folgender Forschungsfragen:

1. Erhöht eine evidenzbasierte Gesundheitsinformation mit der Verknüpfung der Themen Impfschutz und Sepsis die Impfbereitschaft und die Impfquoten von Sepsis-Risikogruppen?
2. Erhöht eine evidenzbasierte Gesundheitsinformation die Kenntnisse von Risikogruppen über Frühwarnsymptome einer Sepsis und das Wissen, dass Sepsis ein Notfall ist?

Als sekundäre Forschungsfragen sollen untersucht werden:

1. Welche evidenzbasierten Informationsformate sind am besten zur Stärkung der gesundheitsbezogenen Wissenskompetenz und des gesundheitsrelevanten Selbstmanagements von Sepsis-Risikogruppen geeignet?
2. Welche Kommunikationskanäle sind für Sepsis-Risikogruppen am besten geeignet, um die impf- und sepsisbezogene Gesundheitskompetenz zu verbessern?
3. Welche individuellen Faktoren beeinflussen Impfbereitschaft und Sepsiswissen von Sepsis-Risikogruppen?

4. Studiendesign und -beschreibung

4.1. Art der Studie

Es handelt sich um eine Interventionsstudie im kontrollierten Design. Die Intervention besteht aus einer multimodalen Informationskampagne, die auf der Basis evidenzbasierter Gesundheitsinformationen das präventions- und früherkennungsrelevante Wissen zur Sepsis sowie die Impfbereitschaft in spezifischen Risikogruppen steigern soll.

4.2. Einschlusskriterien und Rekrutierung

Die Studie besteht aus vier Arbeitspaketen (AP). In AP1 werden Informationsmaterialien entwickelt und vorgetestet, die bei der Durchführung der Informationskampagne (Intervention) im Rahmen von AP2 eingesetzt werden sollen. Bestandteil von AP2 ist außerdem eine begleitende Prozessevaluation. Die Evaluation der Intervention erfolgt im Rahmen von AP3 mittels einer Auswertung von Impfquoten und -inzidenzen gegen Influenza, Pneumokokken, Meningokokken und Hämophilus influenzae in den genannten Risikogruppen, sowie in AP 4 durch eine Befragung von Risikogruppen.

Arbeitspaket 1 (Entwicklung evidenzbasierter Gesundheitsinformationen): Für die Rekrutierung der Befragungsteilnehmer in AP 1 wird ein Dienstleistungsauftrag an ein im Bereich der gesundheitsbezogenen Feldforschung erfahrenes Meinungsforschungsinstitut mit Zugriff auf etablierte Onlinepanels vergeben. Eine Stichprobe mit vordefinierten Anteilen der jeweiligen Risikogruppen wird akquiriert. Es wird angestrebt, dass die Gesamtstichprobe ungefähr die gleiche Zahl an Probanden unter und ab 60 Jahren beinhaltet. Es werden nur Probanden ab 18 Jahren eingeschlossen. Die erhobenen Daten werden vom Dienstleister in anonymisierter Form zur Verfügung gestellt.

Arbeitspaket 2 (Informationskampagne): Die Zielpopulation der Informationskampagne bilden bekannte Sepsis-Risikogruppen, für die es auch eine Impfempfehlung seitens der

Ständigen Impfkommission (STIKO) am RKI gibt: Menschen über 60 Jahre, Schwangere, Menschen mit angeborener oder erworbener Fehlfunktion des Immunsystems (u.a. bei HIV/AIDS), Menschen ohne Milz, Patienten unter Langzeittherapie mit immunsupprimierenden Medikamenten (z.B. wegen rheumatischer Erkrankungen, Autoimmunerkrankungen, schwerer Psoriasis), Menschen mit Krebs, Menschen mit chronischen Erkrankungen des Herzens, der Lunge, der Leber, der Niere oder Diabetes mellitus, Alkohol- bzw. Drogenabhängige. Der Anteil von Menschen an der Gesamtbevölkerung, die o.g. Kriterien erfüllen, beträgt derzeit ca. 34,4%. Für die Modellregionen Berlin und Brandenburg gehen wir von einer Gesamtrisikopopulation von ca. 2,5 Millionen Personen aus.

Arbeitspaket 3 (Messung von Impfquoten und –inzidenzen): Es wird das am RKI etablierte System der Übermittlung und Analyse von Abrechnungsdaten der Kassenärztlichen Vereinigungen (KVen) aus der ambulanten Versorgung genutzt. Daten zu allen Impfleistungen, Vorsorgeuntersuchungen und ausgewählten Krankheitsdiagnosen liegen dem RKI in anonymisierter Form für alle Versicherten der Gesetzlichen Krankenkassen (GKV) seit 2004 vor. Einschlusskriterien für die Identifizierung von Angehörigen der Risikogruppen werden in folgender Weise formuliert: Alter entsprechend der Impfindikationen laut STIKO, ausgesuchte ICD-10 Codes für die relevanten Krankheiten (z.B. J44 für COPD). Aus den KV-Daten gehen keine individuellen GKV-Versichertenzeiten hervor. Daher wird ein Kohortenansatz gewählt, bei dem nur Versicherte eingeschlossen werden, die vor und nach dem entsprechenden Beobachtungszeitraum einen beliebigen Arztkontakt hatten. Dies selektiert nur durchgängig versicherte Personen mit dauerhaftem Wohnsitz im entsprechenden KV-Gebiet. Für Pneumokokken sollen bspw. Versicherte über 60 Jahre sowie alle Altersgruppen mit chronischen Grunderkrankungen analysiert werden. Für Patienten mit Grunderkrankungen wird der Anteil derjenigen, die sich in den letzten 6 Jahren nicht haben impfen lassen – für die also eine erneute Impfindikation besteht – an der Gesamtzahl der Versicherten mit aktueller Impfindikation betrachtet.

Arbeitspaket 4 (Befragung von Risikogruppen): Für die Rekrutierung der Befragungsteilnehmer wird ebenfalls ein Dienstleistungsauftrag an ein Meinungsforschungsinstitut mit Zugriff auf etablierte Onlinepanels vergeben. Eine Stichprobe mit vordefinierten Anteilen der jeweiligen Risikogruppen wird akquiriert. Es wird angestrebt, dass die Gesamtstichprobe ungefähr die gleiche Zahl an Probanden unter und ab 60 Jahren beinhaltet. Probanden werden zu gleichen Teilen in der Interventionsregion Berlin und der Interventionsregion Brandenburg rekrutiert. Es werden nur Probanden ab 18 Jahren eingeschlossen. Die erhobenen Daten werden vom Dienstleister in anonymisierter Form zur

Verfügung gestellt. Es erfolgt ein Vorher-Nachher-Vergleich zu drei Erhebungszeitpunkten (vor, während und am Ende der Informationskampagne).

4.3. Ablauf der Studie

4.3.1. Arbeitspaket 1: Entwicklung der Informationsformate

Die zur Erstellung der Formate benötigte aktuelle beste Evidenz wird durch eine systematische Evidenzsynthese und -bewertung epidemiologischer Daten und klinischer Studien erarbeitet. Die relevantesten Informationen sollen in knapp und schnell erfassbaren Kategorien (z.B. „Wichtigste Warnhinweise für eine Sepsis", „Was ist zu tun", „Wie kann das Risiko gesenkt werden") dargestellt werden. Es werden zwei alternative Informationsformate entwickelt. In **Informationsformat 1** werden die Basisinformationen um eine Visualisierung (Faktenbox mit Icons) ergänzt, in **Informationsformat 2** werden sie ohne Visualisierung dargestellt. Faktenboxen sind visuelle, tabellarische Formate, die Informationen zu Nutzen und Schaden präventiver Maßnahmen (z.B. Population mit Impfung) in absoluten Risiken und auf denselben Nenner adjustiert im Vergleich zu einer Kontrollgruppe (z.B. Population ohne Impfung) darstellen und sich in der Edukation zur Nutzen-Schaden-Bilanz von medizinischen Interventionen als effektiv erwiesen haben [14, 15, 17]. Die entwickelten Informationsformate werden an 20 Personen (je 10 unter/ab 60 Jahre) hinsichtlich ihrer Verständlichkeit pilotiert.

Ziel der anschließenden Vortestung ist es zu ermitteln, welches evidenzbasierte Informationsformat (mit oder ohne Faktenbox) am besten zur Stärkung der gesundheitsbezogenen Risiko-/Gesundheitskompetenz und des informierten Entscheidens (gesundheitsrelevantes Selbstmanagement) geeignet ist. Hierzu wird eine onlinebasierte randomisiert-kontrollierte Studie durchgeführt. Die Probanden werden randomisiert einem der beiden Informationsformate zugeordnet. Die Informationen werden am PC präsentiert. Vor und nach der jeweiligen Intervention mit dem jeweiligen Informationsformat wird den Teilnehmern ein onlinebasierter Fragebogen vorgelegt.

4.3.2. Arbeitspaket 2: Durchführung der Informationskampagne

4.3.2.1. Multimodale Implementierungsstrategie der Kampagne

Um möglichst große Effekte zu erzielen, werden die evidenzbasierten Gesundheitsinformationen über verschiedene Module bzw. Kommunikationskanäle implementiert (s. Abbildung 1). Zu den Multiplikatoren, die durch entsprechende „Letters of intent" (LOI) ihre Bereitschaft zur Unterstützung der Kampagne erklärt haben, gehören a)

wesentliche Akteure im Gesundheitswesen, wie Krankenhausträger, Allgemein- und Fachärzte, Pflege- und Rehaeinrichtungen, Versicherer, KVen, Apotheken, Patientengruppen und Selbsthilfeorganisationen, die durch ihre etablierten Kommunikationskanäle einen guten Zugang zur Risikopopulation haben, und b) Kooperationspartner aus den Bereichen Medien und Außenwerbung.

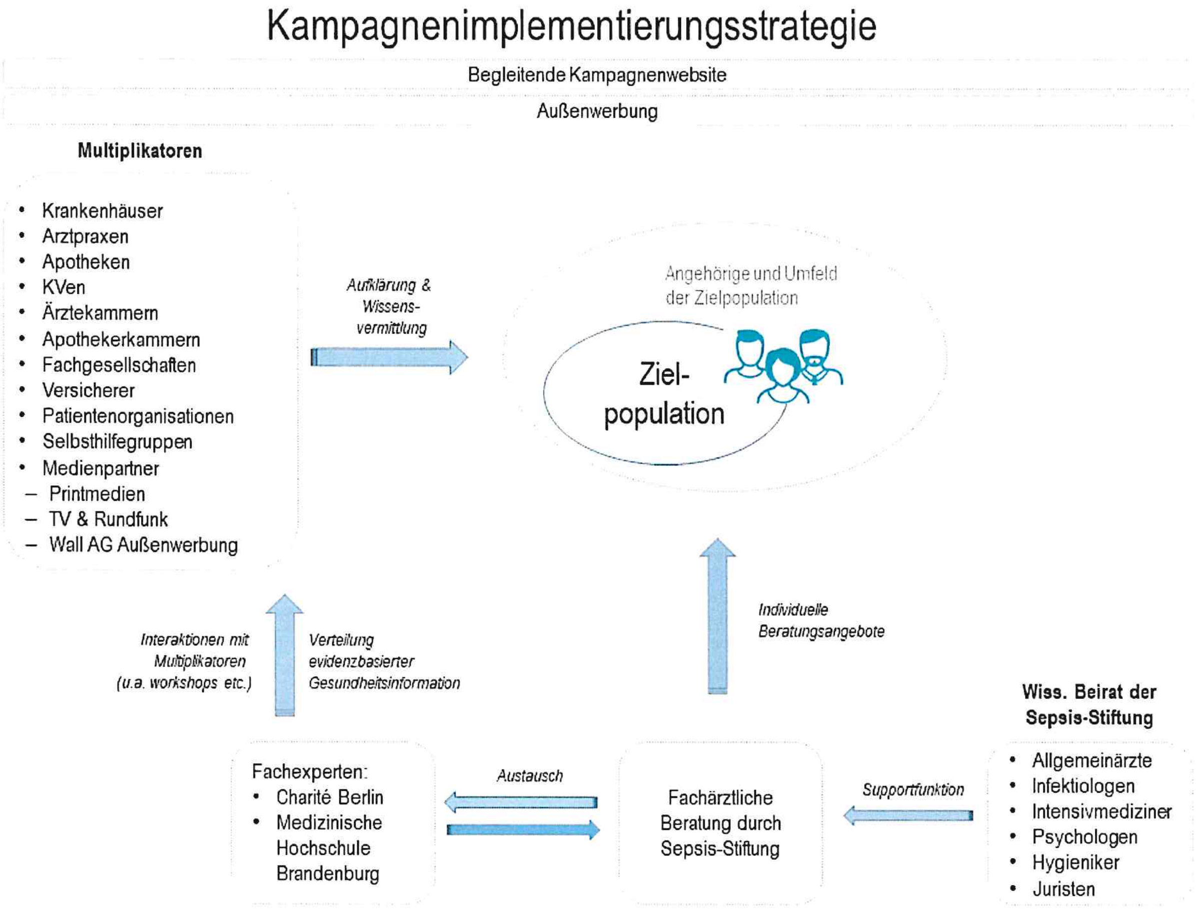

Abbildung 1: Kampagnenimplementierungsstrategie

4.3.2.2 Prozessevaluation

Bei der Intervention handelt es sich in mehrfacher Hinsicht um eine komplexe Intervention (u.a. mehrere interagierende Interventionen, zahlreiche involvierte Gruppen und Organisationen) [24]. Die Replizierbarkeit und Übertragbarkeit auf andere Kontexte ist nur durch eine begleitende Prozessevaluation zu gewährleisten, welche die Umsetzbarkeit und den Erfolg der Implementierung, Wirkmechanismen, sowie Einfluss nehmende Kontextfaktoren erfasst [25]. Abbildung 2 verdeutlicht die Elemente der geplanten Prozessevaluation. Deren Hauptziele sind: a) Unterstützung der Planung der Implementierung, b) Monitoring des Implementierungserfolges und ggf. Anpassung der

Implementierungsstrategie, c) Generierung von Erkenntnissen zu Faktoren für Erfolg oder Misserfolg der Intervention oder einzelner Interventionsteile, um die Übertragbarkeit zu ermöglichen. Die Prozessevaluation begleitet darum das gesamte Projekt von der Planungsphase bis zur abschließenden Ergebnisevaluation und nimmt in SepWiss eine aktive Rolle ein, d.h. Resultate, welche für die Ausgestaltung der Implementierung und den Implementierungserfolg relevant sind, werden frühzeitig kommuniziert und fließen in Veränderungen der Implementierungsstrategie ein [25].. Die Umsetzung der Prozessevaluation erfolgt in geteilter Verantwortung der Studienleitung, der CUB, der MHB, sowie des UKJ unter Einbeziehung der weiteren Projektbeteiligten.

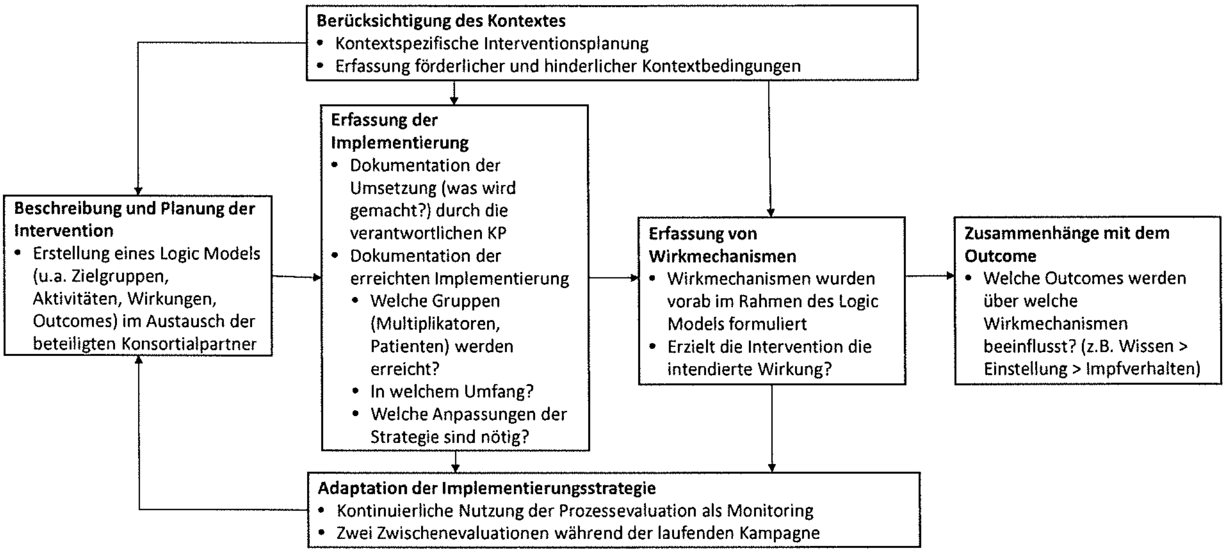

Abbildung 2: Elemente der Prozessevaluation

Während der Planungsphase der Intervention erfolgt die Formulierung und Abstimmung eines Logic Models, das Zwecke, Zielgruppen, Implementierungsaktivitäten, erwartete Wirkmechanismen und Outcomes sowie mögliche Einflussfaktoren systematisiert [26,27]. Auf Basis dieses Logic Models erfolgt die Detailplanung der Implementierungsstrategie. Es dient weiterhin der Planung der Prozessevaluation. Aufgrund des Logic Models werden Indikatoren, Erhebungsstrategien und Erhebungsinstrumente der Prozessevaluation im Detail ausformuliert und zwischen den Projektbeteiligten abgestimmt. Aufgrund der eingeschränkten Ressourcen und zur Gewährleistung der Umsetzbarkeit muss sich die Prozessevaluation auf Schwerpunkte fokussieren [25].

Multiplikatoren werden während und nach Schulungen befragt, die als Teil der Kampagne in den Modellregionen durchgeführt werden. Zusätzlich werden ausgewählte Teilnehmer von

Schulungs- oder Train-the-trainer-Programmen um eine informierte Einwilligung für ein Telefoninterview nach 4 Monaten gebeten. Im Rahmen von leitfadengestützten Interviews werden ihre Erfahrungen mit der Umsetzung der Kampagne (Implementierungserfolg), der wahrgenommene Erfolg (Wirkmechanismen) sowie hemmende und förderliche Bedingungen (Kontext) erfasst, z.B. Einschätzung verschiedener push- und pull-Faktoren: Welche Faktoren führen zu erhöhter Nachfrage nach Impfungen (pull-Faktor)? Welche Faktoren erleichtern die Beratung von Risikogruppen zu Sepsis und Impfung (push-Faktor)? Die erhobenen Daten werden qualitativ ausgewertet und Interviews werden so lange durchgeführt, bis eine theoretische Sättigung eintritt. Quantitative Daten werden mittels adäquater Modelle analysiert, z.B. logistische Regression für binäre Daten. Verantwortlich für diese Erhebungen sind die Charité sowie die MHB.

Die Befragung in AP4 dient nicht nur der Ergebnis- sondern auch der Prozessevaluation. Die erste Erhebungswelle soll die Gestaltung der Implementierungsstrategie unterstützen, indem die häufigsten Quellen und Informationswege für gesundheitsrelevantes Wissen für die befragten Risikogruppen erfasst werden. Auf dieser Basis kann die Implementierungsstrategie der Informationskampagne zielgruppenspezifisch ausgerichtet werden. In der zweiten und dritten Erhebungswelle werden zentrale Aspekte des Implementierungserfolges, sowie bzgl. Wirkmechanismen miterfasst; a) Implementierungserfolg: der Kontakt mit und die Wahrnehmung von Kampagneninhalten, b) Wirkmechanismen: vermittelnde Faktoren bzgl. dem Impf- und Gesundheitsverhalten gemäß etablierter Verhaltensmodelle (Theorie geplanten Verhaltens: z.B. Wissen, Einstellungen, Absicht, Verhalten). Die Ergebnisse der zweiten Erhebungswelle fließen in die zweite Zwischenevaluation der Kampagne ein. Verantwortlich für diese Erhebung ist das UKJ.

4.3.3. Arbeitspaket 3: Evaluierung der Informationskampagne durch Auswertung der Impfquoten in den Risikogruppen

Die Evaluation erfolgt durch einen Vergleich der Entwicklung der altersspezifischen Impfquoten bzw. Impfinzidenzen in ausgesuchten Risikogruppen in der Interventionsregion (Berlin-Brandenburg) im Vergleich mit anderen Bundesländern (Kontrollregion). Erste grobe Schätzungen der Impfquoten vor der Intervention sollen für einzelne Alters- und Risikogruppen durchgeführt werden, um diese bei der Planung und Implementierung der Informationskampagne zu berücksichtigen.

4.3.4. Arbeitspaket 4: Evaluierung der Informationskampagne durch Befragung von Risikogruppen in der Modellregion

Durch die Befragung soll evaluiert werden, ob die Gesundheits- und Risikokompetenz in den betreffenden Risikogruppen durch die Kampagne beeinflusst wurden. Die Befragungsstudie

mit Vorher-Nachher-Vergleich erfolgt zu drei Erhebungszeitpunkten (vor, während und am Ende der Informationskampagne). Der Vergleich der Messungen vor und während der Informationskampagne dient dazu, den Effekt der Kampagne zu ermitteln. Der Vergleich der Messungen während und zum Abschluss der Kampagne untersucht, ob der Effekt der Kampagne sich mit längerer Dauer erhöht. Die Erhebung benötigt voraussichtlich jeweils drei Monate und wird in den Monaten Februar bis April umgesetzt, damit Probanden retrospektiv ihr Impfverhalten in der gerade endenden Grippesaison angeben können.

4.4. Anzahl der zu Befragenden und Biometrie

4.4.1. Arbeitspaket 1

4.4.1.1. Messungen

Primärer Endpunkt der Vortestung ist die validierte, binäre Maßeinheit des „informierten Entscheidens“ nach Marteau [18], welche zwischen informierter und nicht-informierter Entscheidung differenziert. Anhand eines validierten Fragebogens werden drei Schwerpunkte erfasst: (1) spezifische Risiko-/Gesundheitskompetenz, (2) Einstellung basierend auf der Theorie des geplanten Verhaltens bezogen auf sepsisrelevante Impfungen und (3) die finale Impfentscheidung. Die spezifische Risiko- und Gesundheitskompetenz (1) wird über fünf spezifische Fragen zur Nutzen-Risiko-Bilanz der Impfung und der Früherkennung von Sepsis gemessen, und die Gesundheitskompetenz anhand dreier auf das Thema Sepsisprävention und -früherkennung adjustierten Fragen aus der HLS-EU-Subskala „Prävention von Erkrankungen“. Die Risiko-/Gesundheitskompetenz wird anhand eines Cut-off-Wertes (kleiner/gleich 4 = inadäquat; ab 5 = adäquat) unterteilt in „adäquate“ bzw. „inadäquate“ Wissensbasis“ (binärer Endpunkt). Die finale Entscheidung (3) wird als „informiert“ klassifiziert, wenn die Risiko-/Gesundheitskompetenz als „gut“ (Score: mindestens 5 von 8 korrekt) kategorisiert wird, und die Einstellung (positiv [Score: <2,5 von 4]/negativ) mit der finalen Entscheidung (ja/nein) korrespondiert. **Sekundärer Endpunkt** ist die Risiko-/Gesundheitskompetenz (binär, mit Cut-off > 4).

4.4.1.2. Fallzahlkalkulation

Eine repräsentative Befragung bei über 60-Jährigen in Deutschland im Jahr 2017 [3] ergab, dass gegenwärtig nur 17% der Bevölkerung über relevantes Basiswissen zur Sepsis verfügt, welches eine elementare Grundlage für den primären Endpunkt des „informierten Entscheidens“ im Sinne der Definition von Marteau bildet. Der Basiswert für den primären Endpunkt des informierten Entscheidens (dichotomisiert) wird dementsprechend mit 17% angenommen. Die unterschiedlichen Formate der evidenzbasierten Gesundheitsinformation –

die im Rahmen der hier beantragten Vortestung untersucht werden sollen – haben mit Blick auf eine Stärkung des primären Endpunkts in verschiedenen anderen Settings Zuwächse von 12,0% bis 43,5% erreicht [17, 19, 20]. Unter der davon abgeleiteten konservativen Annahme, dass ein absoluter Anstieg des primären Endpunkts durch eine effektive evidenzbasierte Gesundheitsinformation von 20 Prozentpunkte möglich ist, und dass basierend auf den Ergebnissen von Vorstudien die gegenwärtige Basisrate des Sepsis-relevanten Wissens innerhalb der Bevölkerung bei circa 17% liegt [3], werden die hier untersuchten evidenzbasierte Gesundheitsinformation nur dann als effektiv angesehen, wenn im Nachgang der Intervention mindestens ein Anteil von 37% der Befragten eine „informierte Entscheidung" trifft. Der primäre Endpunkt des „informierten Entscheidens" nach Marteau inkludiert, dass die Risiko- und Gesundheitskompetenz der Befragten durch die Intervention mindestens um dieselbe Prozentpunktezahl angestiegen sein muss. Neben einer Bestimmung der generellen Effektivität der beiden Gesundheitsinformationen bzgl. der Stärkung der Gesundheitskompetenz wird in der Vortestung überdies geprüft, ob eine Gesundheitsinformation der anderen überlegen ist. Um als überlegen angesehen zu werden, ist der innerhalb der Vortestung postulierte Anspruch, dass sich die beiden Gesundheitsinformationen in ihrer Wirksamkeit um mindestens 15 Prozentpunkte unterscheiden (52% vs. 37% nach Intervention). Bei Berücksichtigung dieser postulierten Unterschiede und unter Verwendung eines Chi-Quadrat Tests (Signifikanzniveau 5% zweiseitig, Power 90%) werden damit je Interventionsarm 242 Probanden benötigt (nQuery 7.0).

4.4.1.3. Statistische Analysen

Die Analyse bezieht sich auf den primären Endpunkt des informierten Entscheidens und auf den sekundären Endpunkt der spezifischen Risiko-/Gesundheitskompetenz. Der Unterschied der Endpunkte zwischen der Vorher-/Nachher-Messung sowie der beiden Informationsformate wird über Häufigkeitsanalysen mit Konfidenzintervallen erfasst und mittels Fisher's exaktem Test auf Signifikanz geprüft. In Sekundäranalysen werden diese Unterschiede zusätzlich innerhalb der Altersgruppen (<60 vs. ≥60) geprüft.

4.4.1. Arbeitspaket 2

Die Prozessevaluation wird im Laufe des ersten Projektjahres genauer ausgearbeitet, Angaben zur Anzahl der Befragten und zur Messung des Kampagnenerfolgs und zur Beurteilung förderlicher Faktoren werden nachgereicht.

4.4.2. Arbeitspaket 3

4.4.2.1. Messungen

Zur Messung von Impfquoten und Impfinzidenzen wird das am RKI etablierte System der Übermittlung und Analyse von Abrechnungsdaten der Kassenärztlichen Vereinigungen (KVen)

aus der ambulanten Versorgung genutzt [21, 22]. Daten zu allen Impfleistungen, Vorsorgeuntersuchungen und ausgewählten Krankheitsdiagnosen liegen dem RKI in anonymisierter Form für alle Versicherten der Gesetzlichen Krankenkassen (GKV) seit 2004 vor. Interne und externe Validierungen müssen sowohl für die KV-Abrechnungsdaten zu den zugrundliegenden Grunderkrankungen, die eine Person als Risikopatient definieren, erfolgen, als auch für die Impfquote/Impfinzidenz der vier Impfungen (gegen Influenza, Pneumokokken, Meningokokken und Hib) in diesen einzelnen Risikogruppen. Für die externe Validierung kann u.a. auf den Datensatz von Insight Health zurückgegriffen werden, der ebenfalls am RKI verfügbar ist, und Rezeptverordnungen zulasten der GKV beinhaltet. Des Weiteren erfolgt eine Literatursuche zur Identifizierung von Surveys mit Impfquoten in diesen Gruppen.

4.4.2.2. Statistische Analysen

Initial werden für einen Zeitraum vor der Intervention (ca. 5 Jahre) Impfquoten der einzelnen Impfungen in den Risikogruppen (z.B. Patienten mit Asplenie, kardiovaskulären Erkrankungen, Stoffwechselerkrankungen, pulmonalen Erkrankungen, Krebserkrankungen, Immundefizienz) je nach Empfehlung der STIKO berechnet. Zudem werden stratifizierte Analysen in Bezug auf Alter, Geschlecht und Landkreis durchgeführt, um besondere Defizite zu erkennen. Nach Durchführung der Intervention erfolgen Analysen, um mögliche Veränderungen der Impfquoten bzw. Impfinzidenzen als Folge der Intervention zu identifizieren. Dies erfolgt durch einen Vergleich der Trends in Berlin-Brandenburg mit anderen Regionen. Zum Einsatz kommen Regressionsmodelle, wobei der Interventionseffekt durch Analyse von Unterschieden zwischen den Regionen (Interventions- vs. Kontrollregion) und den Zeitperioden (vor vs. nach der Kampagne) untersucht wird.

4.4.3. Arbeitspaket 4: Befragung von Risikogruppen

4.4.3.1. Messungen

Primäre und sekundäre Zielgrößen entsprechen denen unter dem Punkt „Entwicklung und Vortestung der evidenzbasierten Gesundheitsinformationen“ beschriebenen (primäre Zielgröße: Maß des informierten Entscheidens, sekundäre Zielgröße: spezifische Risiko/Gesundheitskompetenz). Als weitere sekundäre Zielgrößen werden erfasst: a) Sepsiswissen (entsprechend eines modifizierten Sepsiswissens-Scores [3], mit Fragen zu Risikofaktoren, Entstehung, Frühsymptomen, Behandlungsoptionen, präventiven Maßnahmen); b) selbstberichtetes Impfverhalten und Impfintention, sowie c) Konstrukte des von einer Expertengruppe der WHO vorgeschlagenen erweiterten theoretischen Modells zum Impfverhalten auf Basis der Theorie geplanten Verhaltens mittels validierter Skalen [23]. Zum ersten Messzeitpunkt werden zusätzlich die häufigsten Quellen und Informationswege für gesundheitsrelevantes Wissen über standardisierte Items erfasst, um auf dieser Basis die

Implementierungsstrategien der Informationskampagne zielgruppenspezifisch ausrichten zu können.

4.4.3.2. Fallzahlkalkulation

Da eine wiederholte Befragung der gleichen Probanden im Rahmen der beschriebenen Stichprobenakquirierung über Panels nicht gewährleistet werden kann, wird für die Fallzahlüberlegungen von unabhängigen Stichproben ausgegangen. Wie für die Entwicklung der Gesundheitsinformationsformate in AP 1 beschrieben, wird von einem Ausgangsniveau der Gesundheits- und Risikokompetenz von 17% ausgegangen. Da im Rahmen einer breiten Kampagne die Probanden nicht alle systematisch erreicht werden können, wird von einer Steigerung um 10% ausgegangen. Für die statistische Überprüfung dieses Unterschiedes werden je Erhebung 379 Probanden benötigt (Chi-Quadrat-Test, Signifikanzniveau 0,05, zweiseitig, Power 90%, nQuery 7.0). Diese Stichprobengröße erlaubt zugleich die Untersuchung komplexer observationaler Fragestellungen durch entsprechende Regressionsmodelle.

4.4.3.3. Statistische Analysen

Unterschiede zwischen den Erhebungen in den primären und sekundären Zielgrößen werden deskriptiv, tabellarisch und grafisch mit Konfidenzintervallen präsentiert. Die Testung auf Unterschiede erfolgt mittels üblicher adäquater Testverfahren entsprechend dem Skalenniveau. Zusätzlich werden theoriegeleitete Fragestellungen, etwa zu vermittelnden oder moderierenden Faktoren zwischen Kampagnenkontakt und Impfentscheidung, durch Einsatz adäquater Modelle analysiert. Konkrete Hypothesen und explorative Fragestellungen werden vor Datensichtung im Rahmen eines Analyseplans spezifiziert.

4.5. Zeitplan

Die Gesamtdauer des Projekts beträgt 36 Monate (August 2020 – Juli 2023). Der Studie liegt der folgende Zeitplan zugrunde:

Name des Arbeitspaketes	Erster Meilenstein	Letzter Meilenstein	Verantwortlich
AP1: Entwicklung von evidenzbasierten Gesundheitsinformationen	11/2020	06/2021	MPI

AP2: Entwicklung, Durchführung und Evaluierung der Informationskampagne	02/2021	06/2023	KF, CUB, MHB, UKJ
AP3: Etablierung der Methodik und Analyse der Impfquoten	11/2020	05/2023	RKI
AP4: Entwicklung der Fragebogen und Befragung in Risikogruppen	11/2020	06/2023	UKJ, IPSOS
Zusammenfassung der Ergebnisse		07/2023	Alle

MPI – Max-Planck-Institut für Bildungsforschung, KF – Konsortialführer (Sepsis-Stiftung), CUB – Charité Universitätsmedizin Berlin, MHB – Medizinische Hochschule Brandenburg, UKJ – Universitätsklinikum Jena, RKI - Robert Koch-Institut

5. Teilnehmende Einrichtungen

Studienleitung/Konsortialführung

Name, Vorname, Titel: Prof. Dr. Reinhart, Konrad
Klinik/Institution: Sepsis-Stiftung
Telefon-Nummer: +49 30 450551415
E-Mail: konrad.reinhart@charite.de

Studienzentrum Charité Universitätsmedizin Berlin (CUB)

Name, Vorname, Titel: Prof. Dr. Spies, Claudia; PD Dr. Hartog, Christiane
Klinik/Institution: Klinik für Anästhesie, Charité Universitätsmedizin Berlin

Tel. +49 30 450 531 089
E-Mail: Christiane.hartog@charite.de, Claudia.spies@charite.de

Studienzentrum Max-Planck-Institut für Bildungsforschung, Berlin (MPIB)

Name, Vorname, Titel: PD Dr. Wegwarth, Odette
Klinik/Institution: Max-Planck-Institut für Bildungsforschung
Tel. +49 30 82 406 695
E-Mail: wegwarth@mpib-berlin.mpg.de

Studienzentrum Medizinische Hochschule Brandenburg (MHB)

Name, Vorname, Titel: Prof. Dr. Neugebauer, Edmund
Klinik/Institution: Medizinische Hochschule Brandenburg
Tel. +49 3391 39141 10
E-Mail: Edmund.neugebauer@mhb-fontane.de

Studienzentrum Robert Koch-Institut (RKI)

Name, Vorname, Titel: PD Dr. Wichmann, Ole
Klinik/Institution: Robert Koch-Institut
Tel. +49 30 18754 3468
E-Mail: WichmannO@rki.de

Studienzentrum Universitätsklinik Jena (UKJ)

Name, Vorname, Titel: Dr. Fleischmann-Struzek, Carolin
Klinik/Institution: IFB Sepsis und Sepsisfolgen, Univ.-Klinik Jena (UKJ)
Tel. +49 3641 9323 146
Email: Carolin.fleischmann@med.uni-jena.de

Name, Vorname, Titel: Dr. Schwarzkopf, Daniel
Klinik/Institution: Univ.-Klinik Jena (UKJ)

Tel. +49 3641 9323 195
Email: Daniel.schwarzkopf@med.uni-jena.de

Name, Vorname, Titel: Prof. Dr. Schlattmann, Peter
Klinik/Institution: Institut für Medizinische Statistik, Informatik und Dokumentation, Univ.-Klinik Jena (UKJ)
Tel. +49 3641 934 130
E-Mail: peter.schlattmann@med.uni-jena.de

6. Datenmanagement

Die zur Erreichung der Studienziele im Rahmen von AP1 und AP4 auszuwertenden Daten werden extern von einem Dienstleister erhoben. Sie werden dem jeweiligen Studienzentrum ausschließlich in anonymisierter Form übermittelt. Ein Rückschluss auf einzelne Befragte ist nicht möglich. Erstellung und Umsetzung eines entsprechenden Datenschutzkonzepts werden vertraglich mit dem Dienstleister geregelt.

Bei den im Rahmen von AP3 auszuwertenden Daten handelt es sich um vertragsärztliche Abrechnungsdaten der Kassenärztlichen Vereinigungen (KVen) in Deutschland. Ein Datenschutzkonzept für die KV-Impfsurveillance durch das RKI inkl. eines positiven Votums des Bundesbeauftragten für den Datenschutz liegt vor. Die auszuwertenden Daten sind anonymisiert.

Für die im Rahmen der Prozessevaluation umgesetzten Befragungen von Multiplikatoren wird vorab ein Datenschutzkonzept erstellt und der Ethikkommission nachgereicht. Die rechtlichen Rahmenbedingungen (DSGVO, BDSG, SGB V und SGB X) werden entsprechend berücksichtigt.

Jede Veröffentlichung von Daten erfolgt ausschließlich in aggregierter und damit anonymisierter Form. Angaben über einzelne Patienten oder Erhebungszentren werden nicht veröffentlicht oder an Dritte weitergegeben.

7. Ethische Belange, gesetzliche und administrative Regelungen

7.1. Deklaration von Helsinki und Gute klinische Praxis

Die Studie wird gemäß den ethischen Grundsätzen durchgeführt, die ihren Ursprung in der Deklaration von Helsinki haben. Die jeweils aktuelle Version der Deklaration wird beachtet. Die Empfehlungen der Guten Klinischen Praxis, gültig seit dem 17.1.1997, werden, sofern zutreffend, berücksichtigt.

7.2. Ethik-Kommissionen

Das Studienprotokoll wird mit den erforderlichen weiteren Unterlagen der zuständigen Ethik-Kommission des Studienleiters mit der Bitte um Bewertung vorgelegt. Die Studie kann erst nach zustimmender Bewertung der Ethik-Kommission beginnen.

7.3. Nachträgliche Änderungen

Änderungen oder Ergänzungen des Studienprotokolls können nur vom Studienleiter veranlasst und autorisiert werden. Über Änderungen des Studienprotokolls wird die Ethikkommission informiert. Ggf. wird erneut die zustimmende Bewertung eingeholt. Bewertungspflichtige Änderungen dürfen nicht vor der Entscheidung der Ethikkommission umgesetzt werden.

Änderungen der von der Ethik-Kommission zustimmend bewerteten Studie sind:

- Auswirkungen auf die Sicherheit der betroffenen Personen,
- zusätzliche Datenerhebungen oder Auswertungen, die eine Änderung der Patienteninformation und/oder -einwilligung erfordern,
- die Auslegung der wissenschaftlichen Dokumente, auf die die Studie gestützt wird, oder die wissenschaftliche Aussagekraft der Studienergebnisse zu beeinflussen,
- die Art der Leitung oder Durchführung der Studie wesentlich zu verändern.

8. Finanzierung

Die Studie wird vom Innovationsfonds für Versorgungsforschung des Gemeinsamen Bundesausschusses für eine Laufzeit von 36 Monaten gefördert (FKZ 01VSF19020).

9. Abschlussbericht und Publikation

Die Veröffentlichung der Studienergebnisse erfolgt unabhängig davon, wie die Ergebnisse ausfallen.

Eine Kopie des Abschlussberichtes bzw. der Publikation wird der Ethikkommission vorgelegt.

10. Literatur

1. World Health Organisation Executive Board (EB140/12). *Improving the prevention, diagnosis and clinical management of sepsis.* 2017 2017/06/20]; Available from: http://apps.who.int/gb/ebwha/pdf_files/EB140/B140_12-en.pdf.

2. Fleischmann-Struzek, C., et al., *Challenges in Assessing the Burden of Sepsis and Understanding the Inequalities of Sepsis Outcomes between National Health Systems - Secular Trends in Sepsis and Infection Incidence and Mortality in Germany* Intensive Care Med 2018. **44**(11): p. 1826-1835.

3. Eitze, S., et al., *Determinants of sepsis knowledge: A representative survey of the elderly population in Germany.* Crit Care, 2018. **22**(1): p. 273.

4. Bonten, M.J., et al., *Polysaccharide conjugate vaccine against pneumococcal pneumonia in adults.* N Engl J Med, 2015. **372**(12): p. 1114-25.

5. Darvishian, M., et al., *Effectiveness of seasonal influenza vaccine in community-dwelling elderly people: a meta-analysis of test-negative design case-control studies.* Lancet Infect Dis, 2014. **14**(12): p. 1228-39.

6. Niehues, T., et al., *Impfen bei Immundefizienz.* Bundesgesundheitsblatt - Gesundheitsforschung - Gesundheitsschutz, 2017. **Volume 60**(Issue 6, pp 674–684).

7. Danai, P.A., et al., *The epidemiology of sepsis in patients with malignancy.* Chest, 2006. **129**(6): p. 1432-40.

8. Gingo, M.R. and A. Morris, *HIV Infection and Severe Sepsis: A Bitter Pill to Swallow.* Crit Care Med, 2015. **43**(8): p. 1779-80.

9. Seymour, C.W., et al., *Time to Treatment and Mortality during Mandated Emergency Care for Sepsis.* N Engl J Med, 2017. **376**(23): p. 2235-2244.

10. Burrell, A.R., et al., *SEPSIS KILLS: early intervention saves lives.* Med J Aust, 2016. **204**(2): p. 73.

11. Torio, C.M. and B.J. Moore, *National Inpatient Hospital Costs: The Most Expensive Conditions by Payer, 2013: Statistical Brief #204*, in *Healthcare Cost and Utilization Project (HCUP) Statistical Briefs.* 2016: Rockville (MD).

12. Fleischmann, C., et al., *Hospital Incidence and Mortality Rates of Sepsis.* Dtsch Arztebl Int, 2016. **113**(10): p. 159-66.

13. Schaeffer, D., et al., *Gesundheitskompetenz der Bevölkerung in Deutschland: Ergebnisbericht.* 2016, Universität Bielefeld, Fakultät für Gesundheitswissenschaften: Bielefeld.

14. Schwartz, L.M., S. Woloshin, and H.G. Welch, *The drug facts box: Providing consumers with simple tabular data on drug benefit and harm.* Medical Decision Making, 2007. **27**: p. 655–662.

15. McDowell, M., et al., *Tabular and icon fact box formats facilitate comprehension and increase knowledge compared to standard health pamphlets: a randomized trial.* Medical Decision Making, 2019. **39**(1): p. 41–56.

16. Gaissmaier, W., et al., *Numbers can be worth a thousand pictures: individual differences in understanding graphical and numerical representations of health-related information.* Health Psychology, 2012. **3**(31): p. 286–296.

17. Muehlbauer, V., et al., *Alternative package leaflets improve people's understanding of drug side effects - A randomized controlled exploratory survey.* PLoS One, 2018. **13(9):e0203800.**

18. Marteau, T.M., E. Dormandy, and S. Michie, *A measure of informed choice.* Health Expectations, 2001. **4**(4): p. 99–108.

19. McDowell, M., et al., *Tabular and icon fact box formats facilitate comprehension and increase knowledge compared to standard health pamphlets: a randomized trial.* Medical Decision Making, 2019. **39**(1): p. 41.

20. Steckelberg, A., et al., *Effect of evidence based risk communication on "informed choice" in colorectal cancer screening: randomised controlled trial.* British Medical Journal, 2011. **342**: p. d3193.

21. Betsch, C., et al., *Increasing influenza and pneumococcal vaccine uptake in the elderly: study protocol for the multi-methods prospective intervention study Vaccination60.* BMC Public Health, 2018. **18**(1): p. 885.

22. Rieck, T., et al., *Aktuelles aus der KV-Impfsurveillance – Impfquoten ausgewählter Schutzimpfungen in Deutschland.* Epid. Bull. 2018;1:1 – 14, 2018.

23. World Health Organization, *Barriers of influenza vaccination intention and behavior – A systematic review of influenza vaccine hesitancy 2005 – 2016 October.* 2016: Geneva.

24. Craig, P., et al. *Developing and evaluating complex interventions: the new Medical Research Council guidance.* British Medical Journal, 2008. **337**.

25. Moore, GF., et al. *Process evaluation of complex interventions: Medical Research Council guidance.* BMJ : British Medical Journal, 2015. **350**.

26. U.S. Department of Health and Human Services Centers for Disease Control and Prevention. Office of the Director. Office of Strategy and Innovation. *Introduction to program evaluation for public health programs: A self-study guide.* Atlanta, GA: Centers for Disease Control and Prevention; 2011.

27. Goeschel, CA., et al. *Using a logic model to design and evaluate quality and patient safety improvement programs.* Int J Qual Health Care, 2012. **24**(4):330-7.
